# Evaluating implementation of a hospital‐based cancer registry to improve childhood cancer care in low‐ and middle‐income countries

**DOI:** 10.1002/cam4.70125

**Published:** 2024-09-09

**Authors:** Melissa R. Maas, Allison Yang, Michele A. Muir, James B. Collins, Courtney Canter, Gevorg Tamamyan, Inam Chitsike, Francine Kouya, Kim Hoa Nguyen, Alia Ahmad, Ana Patricia Alcasabas, Yi‐Jin Gao, Kimberly J. Johnson, Gia Ferrara, Nickhill Bhakta, Benyam Muluneh

**Affiliations:** ^1^ Division of Pharmacotherapy and Experimental Therapeutics University of North Carolina Eshelman School of Pharmacy Chapel Hill North Carolina USA; ^2^ GSK Durham North Carolina USA; ^3^ North Carolina Translational and Clinical Sciences Institute Chapel Hill North Carolina USA; ^4^ Pediatric Cancer and Blood Disorders Center of Armenia Yeolyan Hematology and Oncology Center Yerevan Armenia; ^5^ Department of Hematology and Pediatric Oncology Yerevan State Medical University Yerevan Armenia; ^6^ Parirenyatwa Group of Hospitals Harare Zimbabwe; ^7^ Mbingo Baptist Hospital Cameroon; ^8^ Hue Central Hospital Hue Vietnam; ^9^ University of Child Health Sciences, The Children's Hospital Lahore Pakistan; ^10^ University of Philippine—Philippine General Hospital Manila Philippines; ^11^ Shanghai Children's Medical Center Shanghai China; ^12^ Brown School at Washington University in St. Louis St. Louis Missouri USA; ^13^ Department of Global Pediatric Medicine St. Jude Children's Research Hospital Memphis Tennessee USA

**Keywords:** cancer outcomes, cancer registry, clinical cancer research, pediatric cancer

## Abstract

**Purpose:**

Cancer is a leading cause of global childhood mortality, affecting 400,000 children annually. While treatable with modern therapies, children living in low‐ and middle‐income countries (LMICs) have limited access to care and lower survival rates. Hospital‐based cancer registries (HBCRs) collect detailed patient information to critically evaluate and evolve care. The St. Jude Global Childhood Cancer Analytics Resource and Epidemiological Surveillance System (SJCARES) is a cloud‐based HBCR network facilitating quality data collection of pediatric cancer. Wide variation in the success of implementation has warranted further research into the implementation approach, to create a sustainable and adaptable HBCR in LMICs.

**Methods:**

Seven of 89 sites using the SJCARES registry were selected, stratified by global region and stage of implementation. Semi‐structured interviews were conducted with key groups (clinicians, administrators, data clerks) using an interview guide developed from the Consolidation Framework for Implementation Research (CFIR). Interviews were conducted via a video‐telephone software program and transcribed by a transcription service. Transcripts were thematically coded using rapid qualitative analysis.

**Results:**

A total of 18 participants (11 clinicians, 4 administrators, 3 data clerks) were interviewed. Several barrier themes were identified, including: difficulty integrating the registry into existing workflow; lack of resources; lack of government or administrative support; and damaged, misplaced, or illegible medical records. Facilitator themes were identified, including: internal support for the registry; clear and extensive training; and dedicated support staff.

**Conclusion:**

Interviewed participants identified key barriers and facilitators to the implementation of the SJCARES registry across multiple phases. We plan to use these results to develop targeted implementation strategies including a readiness assessment tool to help guide more successful implementation of the SJCARES registry and other HBCRs in LMICs.

## 1 INTRODUCTION

Childhood cancer is curable for those who have access to modern treatments and robust supportive care, with greater than 85% of children expected to survive beyond 5‐years.^1^, ^2^, ^3^ However, for the nearly 90% of children who live in low‐ and middle‐income countries (LMICs), multiple factors including financial hardship, limited access to treatment, and reduced quality care, have contributed to an estimated 20%–30% global 5‐year net survival worldwide.^2^, ^4^

To address this gap, in 2018 the World Health Organization (WHO), with the support of St. Jude Children's Research Hospital, launched the Global Initiative for Childhood Cancer (GICC). To support implementation of the GICC, the WHO developed the *CureAll* Framework to ensure that comprehensive cancer control policies are developed in each country. As part of this framework, the fourth pillar, or “e” in the *CureAll* acronym, stands for “evaluation and monitoring with information systems to improve outcomes.”^5^ A key strategy under the pillar is investment in both population‐based cancer registries (PBCRs) and hospital‐based cancer registries (HBCRs).

Cancer registries are an essential cancer control tool for understanding improvements in treatment through the collection of patient information, as they provide monitoring tools to track patient outcomes.^6^ PBCRs are the mainstay of global cancer registration, collecting information on all patients within a defined geographical location. Usually implemented and maintained by government agencies, PBCRs provide health services level data to determine incidence trends over time, monitor survival outcomes through linkages with vital registries, and identify potential risk factors. In contrast, HBCRs are managed at the facility level and contain data from all patients treated within a specific hospital or hospital system.^7^ HBCRs provide data on cancer occurrence, diagnosis, treatment, outcomes, and follow‐up for each patient. This data provides insights into the quality of care at a health services level. While HBCRs cannot provide information on cancer burden in a population, they are a valuable resource that can also serve as a primary data source for PBCRs.^8^

Previous registry efforts in LMICs around the world have been inconsistent and individualized, with varying degrees of success. PBCRs currently cover nearly all pediatric cancer patients in North America. However in Africa, Latin America, and Asia, only 2%, 3%, and 6.3% of the population pediatric cancer patients are registered, respectively.^9^, ^10^ An exploration of the current climate of PBCRs in Africa and Asia revealed that there are no high‐quality national PBCRs in Africa, and very few in Asia.^9^ Many countries on these continents lack both national and regional PBCRs.^9^ Currently 20% of Asian countries have PBCRs, with only four countries (Korea, Taiwan, Japan, and Singapore) having a majority of patients covered by PBCRs.^9^

There is robust guidance from global agencies such as the International Agency for Research on Cancer (IARC) and WHO regarding best practices for the implementation of PBCRs in LMICs.^9^ However, guidance for implementing HBCRs worldwide does not exist. There are a few examples of successful HBCRs for tracking adult cancer patients in LMICs, such as in Latin America.^10^, ^11^ But despite these data, there remains a lack of information in the literature on HBCRs which include pediatric cancer data for resource‐limited settings.

In recognition of this gap, the St. Jude Department of Global Pediatric Medicine developed the St. Jude Global Childhood Cancer Analytics Resource and Epidemiological Surveillance System (SJCARES) registry platform.^12^ The SJCARES registry was designed as an intuitive, cloud‐based platform to facilitate high‐quality hospital‐based data collection in LMICs. Over the past 2 years, however, implementation of the registry network has been uneven with rapid adoption at some hospitals and slower adoption among others. The SJCARES registry aims to integrate previous registry efforts into a more sustainable, comprehensive, and universal database, with reliable storage of data. This study's objective is to enhance future implementation of the SJCARES registry by refining the pre‐implementation process through identifying barriers and facilitators.

## 2 METHODS

### 2.1 Study design

Semi‐structured interviews were conducted to identify barriers and facilitators to implementation of the SJCARES registry. The research team designed and pilot‐tested an interview guide made by adapting questions from the Consolidated Framework for Implementation Research (CFIR) interview guide tool.^13^ The interview questions were then refined and adapted to LMICs to create a comprehensive interview guide to assess the adoption and implementation of the SJCARES registry (see Supplemental—Data S1). This study was approved by both the University of North Carolina and St. Jude Children's Research Hospital Institutional Review Boards (#22‐0226).

### 2.2 Study setting and participants

At the time of the study initiation in September 2021, a total of 89 hospitals from over 40 countries were affiliated with the St. Jude Global Alliance and had signed SJCARES registry data use agreements. From that group, over 20 hospitals completed the pre‐defined onboarding process. To support study interpretation, the onboarding process was categorized into stages to describe progress toward implementation: phase 1 (onboarding and training), phase 2 (pilot testing and trialing), phase 3 (data collection), and phase 4 (data utilization and review).

All 89 hospitals working toward registry implementation were categorized by global region and stage of implementation. After stratifying by geographic diversity and phase of onboarding, a subset of participating SJCARES registry site directors were randomly invited to attend a webinar to learn more about the study. A random number generator was utilized to select among sites in the same geographic region and phase of onboarding. Directors interested in participating were asked to identify additional participants such as hospital administrators, healthcare providers, and data registrars responsible for data entry. Using the contact information provided, researchers sent email invitations to potential participants who were treating childhood cancers and heavily involved in the registry. Sites were excluded if they were in a state of personnel transition, current internal or global conflict, unresponsive after three contact attempts, or required interpretation services.

### 2.3 Data collection

Semi‐structured interviews were conducted via a video‐telephone software program from August to November 2022. Written and verbal consent was obtained from all participants before scheduling interviews. The content of the interview guides was written and conveyed in English. Interview audio recordings were deidentified, transcribed, and reviewed for accuracy.

### 2.4 Rapid qualitative analysis

Transcribed interviews were analyzed using rapid qualitative analysis.^14^ After completion of the analysis, the data was aggregated into a summary matrix organized by thematic domain and participant group. Findings were synthesized into intrapersonal, interpersonal, and organizational‐level factors to identify the level of influence for each. The summary matrix represents a compilation of perceived barriers and facilitators to implementing the SJCARES registry. After the rapid qualitative analysis, identified CFIR barriers were mapped against the Expert Recommendations for Implementing Change (ERIC) project to identify practical evidence‐based implementation strategies that are known to mitigate the identified barriers.^15^ The ERIC results were then compared to the pre‐study implementation strategies employed by the SJCARES team to identify new opportunities for intervention.

## 3 RESULTS

### 3.1 Participant characteristics

Of the 13 selected SJCARES registry sites, 9 site directors responded, attended the webinar, and provided names and contact information for participants from their site. A total of 18 participants across 7 sites agreed to participate in semi‐structured interviews. Of the 7 participating sites, 1 was in phase 2; 2 were in phase 3; and 4 were in phase 4. Of the 18 total participants: 11 were clinicians, 4 were administrators, and 3 were data clerks. Table 1 displays the centers selected, the phase of implementation, and participants that completed semi‐structured interviews. Table 2 presents barriers and facilitators mapped to the relevant CFIR construct. Table 3 includes pre‐study support services provided by the SJCARES registry team and maps potential ERIC project‐based strategies to identified barriers to enhance implementation.

**TABLE 1:** Characteristics of interview participants.

Center name	Country	Phase	Participants
Pediatric Cancer and Blood Disorders Center of Armenia	Armenia	Phase 4	Four Clinicians
Mbingo Baptist Hospital	Cameroon	Phase 3	One Clinician
			One Administrator
Shanghai Children's Medical Center	China	Phase 2	One Administrator One Data Clerk
University of Child Health Sciences, The Children's Hospital	Pakistan	Phase 4	One Clinician
University of Philippine—Philippine General Hospital	Philippines	Phase 4	One Clinician One Data Clerk
Hue Central Hospital	Vietnam	Phase 2	Four Clinicians
Parirenyatwa Group of Hospitals	Zimbabwe	Phase 4	Two Administrators

*Note*: This table displays the country the hospital is in, the phase of implementation of the SJCARES registry the center is in, and the number of participants interviewed at each site. Each phase is described as follows: phase 1 onboarding and training, phase 2 pilot testing and trialing, phase 3 data collection, and phase 4 data utilization and review.

**TABLE 2:** Key barriers and facilitators to implementation of the SJCARES registry identified with aligned consolidated framework for implementation research (CFIR) construct and quotes.

Key themes	CFIR domain (construct)	Illustrative quotes
Difficulty integrating the registry into existing workflow	Outer setting (partnerships and connections), inner setting (characteristics—knowledge)	“In our hospital, we did not have any hospital‐based cancer registry. We did have our data, which was on registers, mainly. Then sometimes on the Excel sheets, but, really, not all the data on Excel sheets just maybe some patchy data in the computer…” [Clinician 06]
Lack of resources		
Financial	Inner setting (available resources; characteristics—self‐efficacy), characteristics of systems (resource source; external funding agent priorities)	“Every month on the first date of the month, that the person from Dubai he send me that amount to my account… The other person… sent before 10th of that month… “If he gives the money, okay. If he doesn't give me the money, maybe I have to find some other person… These were the, I think, three biggest challenges, monetary incentive for data entry specialist, and to find somebody who will be the data entry specialist, how to sustain the monetary incentive, from where we will be getting…” [Clinician 06]
Personnel		“The core team is very small. We have tried to get other people to help with the data capturing, and they've not been forthcoming mostly because there's no compensation for that extra work for them. We don't have any nurses that work on the unit participating in the registry. We don't have any of the doctors.” [Data Clerk 05]
Time		“We have many patients, so we spend a lot of time for the patients. Now if we start to put the data, so we have a struggle. We have to limit our time to put the data… I think that the most significant barrier is the workload. We don't have many doctors working in the department, so the workload here is very heavy and we have to work OT a lot of time.” [Clinician 01–04]
Technology		“We have only just one laptop that they are using. If it's crashing, you know? We don't even have a desktop.” [Clinician 11] “Currently, since the personnel who are working on the registry are the ones that are providing their own like laptops to do the data capturing, or even the monitoring. If any of them leaves and doesn't … over, that means we would lose some data.” [Data Clerk 05]
Lack of hospital administration and governmental support	Inner setting (roles—implementation leaders; culture—leadership commitment); characteristics of systems (external funding agent priorities)	“the administration should own it and should support it. I know that they have so many other things to do that they don't have extra staff to give us. They don't have extra money to pay for that staff… Maybe people will accept the importance of this registry, and maybe they start owning it. At the moment, they are not in the mood of owning it. Because if they want to own it, they should have given us some stuff out of maybe 4,000 or maybe 3000 workers. At the moment, they are not.” [Clinician 06]
Training or mentorship is not sufficient	Inner setting (access to knowledge and information)	“For onboarding, we had the resources we needed. You have the modules on Cure4Kids, and you can just follow through them. Once we got to the Trial Master, there was not enough support to enter a patient in terms of what exactly you were supposed to do. We got three test cases, and without prior experience of using the register—the registry, we were supposed to put in our information. Then after that, we were supposed to have a meeting. When we eventually did have the meeting, we realized that we'd failed … The reason being no one had walked us through how to use the registry” [Data Clerk 05]
Records damaged, hard to find, or difficulty reading handwriting	Implementations process (planning—assessing context)	“The hard thing in my country was about with scanning the document. That process took so much time, frankly speaking. I do think that the hard thing is this. Otherwise, I do think that it's possible to do in all developing countries. You just have to have access to this medical records.” [Clinician 05] “Maybe for me, it's very important to have electronical data because, as I mentioned before, the main problem, especially from me, is the hand‐written medical report. Sometimes it impossible to read. It took a lot of time because the process searching necessary information takes more time. It can take, for example, two hours for one medical record. If we would have electronical medical report, it will win it, I think” [Clinician 07]
Difficulty of collaboration	Outer setting (partnerships and connections)	“The challenge with the registry is the resources because it needs a lot of resources. It needs a lot of training. It needs a lot of collaboration, even with other hospitals, because we might be a treatment center, but many people get a diagnosis of cancer that sometimes will never reach this treatment centers. Also, some pathology laboratories function on their own and they're not a cancer treatment center, so they will have their information as a pathology laboratory. With this collaboration, we will be able to mesh this information and also to be able to take care of duplicates.” [Administrator 04]
Concerns for future of data	Inner setting (structural characteristics)	“people are very fearful that data will be taken in the custody of one person and that person will manipulate whole data and he will publish hundred articles on her own name and nobody will know about that there are five or seven consultants who are working. I think this is one of the biggest fear in their minds.” [Clinician 06] “One thing I still a little bit worry about is the durability of this program… You begin this registration program, but how long will it last forever, 10 years, 5 years, or 20 years? If we input our patient data into the registration program…maybe someday you decided to close the registration program in your global, so how do we deal with this database… How can we take out all these patient data?” [Administrator 07, Data Clerk 04]
Cultural considerations and language barriers	Inner setting (structural characteristics)	“For example… we named this medicine as biopsy, but in database, biopsy and the microscope pick examination are two separate options, and that cause some misunderstanding, so I think there will be some other problems for other countries…” [Administrator 07, Data Clerk 04] “We are quite confident using English or Russian. I don't know, at the beginning stage it was not easy. Okay, you have the English as a second or third language…Our company is quite small but to have an Armenia version.” [Clinican 12]
High internal support and motivation	Inner setting (culture—relative priority)	“The reason I would like to implement SJCARES Registry because, as you know, in my hospital in order to treatment we should do some research. From some research we can know the quality of treatment and we would like to improve. Without any data we cannot say anything.” [Clinician 01–04]
Dedicated support staff	Inner setting (culture—leadership commitment; roles—implementation leaders)	“I'm a clinician. I'm very, very busy, but I have a good nurse. I think … He's really, really good at putting everything together, discussing with the administration, and he can convince. Truly, I have his support, even when I go through the training, and I have something I don't understand or—yes—sometimes just ask, “How do we do this one? I don't really understand what it wants here,” I also have that support.” [Clinician 11]
Clear and extensive training courses	Inner setting (access to knowledge and information)	“she [previous data clerk] said that the training was actually nice. It helped her a lot better understand the registry… and she can also go back to check the site or the modules to help her answer the information in the registry.” [Data Clerk 02] “The training course that they do online is, for me, is perfectly what they need. It is not so extensive, but is providing the data entry specialist and also the monitor, the local monitor, with the necessary information to understand because for the data entry specialist, learning about diagnosis, treatment, and outcomes is important, but for the pediatric oncologist, understanding why demographics is going to be first, and then diagnosis, and then treatment, and also outcome is just to put together all the knowledge that is required to have a useful and accurate registry.” [Administrator 05]
External support	Outer setting (policies and incentives), characteristics of systems (external funding agent priorities), outer setting (partnerships and connections)	“They all help us. They all encourage us—PSPO [Pakistan Society of Pediatric Oncology], our organization—that we should have registries, we should improve, we should collaborate with St. Jude and with each other. That was one of the strengths that they encouraged that we should have registries, we should have more data, more research. That was one of the strengths. It helped us to continue on it.” [Clinician 06]
Registry is easy to use	Characteristics of systems (external funding agent properties; resource source)	“It is easy to organize, so the data related, data sharing difficulties, there are not that kind of stuff. It is easy to take medical histories to get whatever you want, and the information to put in the portal.” [Clinician 12]
Good organization of medical records	Implementations process (planning—assessing context)	“We also have a decent records room where you can easily find files when you need them. If the parents had to go back, open their folders, then we wouldn't be able to capture anything. When you go into our records room, you can find what you're looking for.” [Data Clerk 05]

*Note*: This table summarizes the barriers and facilitators that were identified by participants. Each barrier and facilitator are categorized with the CFIR domain, and sub‐identified by CFIR construct. Sample quotes are included as examples for each barrier and facilitator.

**TABLE 3:** Expert recommendations for implementing change (ERIC) strategies to address CFIR barriers.

Barrier	CFIR domain (construct)	Pre‐study implementation strategies	Proposed implementation strategies (ERIC)
Difficulty integrating the registry into existing workflow	Inner setting—structural characteristics Inner setting—compatibility	Creation of a 7‐point implementation workflow	Conduct cyclical small tests of change/audit and provide feedback Develop a formal implementation blueprint (process maps, SOP)
Lack of resources (financial, personnel, time, technology)	Inner setting—available resources Intervention characteristics—cost	Pilot funds available on limited basis for St. Jude Global Alliance members Standard data use agreements used by all sites and transparently included on website Letters of support for grants by SJCARES HBCR team	Develop resource sharing agreements (between departments) Access new funding (advocacy, philanthropy, grants) Obtain formal commitments (contracts for stakeholders involved)
Lack of hospital administration and governmental support	Inner setting—leadership engagement Process—external change agents	Meetings and presentations by SJCARES leadership to hospital administrators and/or ministries of health officials Key materials (data use agreements, educational curriculum, data standards) included on website	Identify and prepare champions/recruit, designate, and prepare for leadership Build a coalition (multidisciplinary committee including leaders)
Training or mentorship is not sufficient	Inner setting—access to knowledge and information	Creation of training cohorts between multiple onboarding teams in a country or region who work together on a timeline and are provided regular “office hours” during onboarding process	Identify and prepare champions/provide local technical assistance (local expert to answer IT or clinical questions related to the registry) Conduct ongoing training/make training dynamic (onboarding + long term, guided practice activities during training, provide fact sheets) Organize clinician implementation team meetings
Records damaged, hard to find, or difficulty reading handwriting	Process—planning	N/A	Change physical structure and equipment (consider simplified documentation, electronic documentation, scanned copies of charts)
Difficulty of collaboration	Inner setting—networks and communications	N/A	Organize clinician implementation team meetings Conduct ongoing training
Concerns for future of data governance	Inner setting—culture	Meeting and presentations by SJCARES leadership to hospital administrators and/or ministries of health officials	Conduct educational meetings (to discuss data ownership and purpose of the registry)
Cultural considerations and language barriers	Inner setting—culture	Discussions with local teams on ad hoc basis	Conduct local needs assessment Tailor strategies

*Note*: This table was created using the ERIC Strategies Tool, which incorporates strategies selected by experts to address specific CFIR barriers.

### 3.2 Barriers

A total of nine barrier themes were identified through interviews with participants and are described below. Quotes from participants are included in Table 2.

#### 3.2.1 Difficulty integrating the registry into existing workflow

The majority of participants with no prior HBCR experience identified difficulties implementing the registry. These difficulties included inability to tailor current workflows to include data collection, causing sites to be unable to progress.

#### 3.2.2 Lack of resources

Participants across all groups reported insufficient resources in four key areas: funding, personnel for data entry, time, and technology. Consistently across all sites, lack of funding was the most difficult barrier to address. All groups, especially administrators, noted that limited financial resources hindered hiring of additional staff for the registry and purchasing necessary equipment. In some cases, external funding is available, but administrators and clinicians voiced concerns about these donations' sustainability.

Without financial support, participants rely heavily on volunteer work that is not always forthcoming. And the lack of dedicated data clerks or staff to help with the registry results in delays.

Participants across all groups described how busy schedules, long working hours, and various clinical duties make it challenging to dedicate time to the registry. Clinicians reported spending most of their time seeing patients and an increasing workload since joining the registry due to physician shortages, patient volume, and the COVID‐19 pandemic.

The majority of participants described how access to reliable technology is essential for training and data entry. There is limited access to computers, and some computers have no backup. These computers often crash, which results in slower data input. Many participants reported needing to use a personal laptop to access the registry. This could lead to issues with data loss if they leave their position or do not transfer the data properly.

#### 3.2.3 Lack of hospital administration and governmental support

Many governments prioritize monitoring infectious diseases and other prevalent non‐communicable diseases, instead of childhood cancers. Most participants commented on the need to persuade hospital administrators and government officials for permission to initiate implementation of the registry at their center. Participants across all groups expressed difficulty gaining initial and continued support from these groups. Clinicians and data clerks indicated administrators may not understand the importance of the registry, and thus do not allocate necessary resources.

#### 3.2.4 Records damaged, hard to find, or difficulty reading handwriting

Reading medical records and importing documents into computer systems is a time consuming and labor‐intensive process. Many institutions in LMICs do not have access to electronic medical records. Additionally, clinicians and nurses may be too busy to document the requisite data clearly and properly into the patient's protocol booklet, resulting in missing data in the registry. Participants advocated for implementing electronic medical record systems at their hospitals to reduce time spent searching for necessary information in paper charts and deciphering handwriting.

#### 3.2.5 Training or mentorship is not sufficient

Several participants shared that the training course for onboarding was helpful, but there was a lack of support during the data entry portion of the training course. St. Jude provides test cases for participants to practice entering data into the registry. Participants reported these cases were difficult to complete, and there was minimal guidance on how to handle issues encountered during data entry.

#### 3.2.6 Difficulty of collaboration

It was noted that physicians may not fully understand the importance of the registry, resulting in the perception that entering data into the registry is duplicative work on top of documenting patient information in the medical record. Participants reported collaboration with other hospitals and laboratories is required for complete data entry. This requires constant follow‐up by data clerks to ensure information is being input by the other sites. However, political crises and physical roadblocks impede this collaboration and cause delays in data entry.

#### 3.2.7 Concerns for future of data

Participants across all groups reported that members of their department are hesitant to use the registry due to concerns about the future availability of data, where patient data is stored, and who has access to that data. If the registry is closed in the future, administrators worry about losing access to their patient data. So many also enter this data in another location, increasing the burden of data entry.

#### 3.2.8 Cultural considerations and language barriers

Data clerks expressed difficulty with translating medical terminology, especially when it differs from the options available in the registry. In some cases, sites have been able to adapt to using another language available in the registry such as English or Russian, but this still presents opportunities for errors. It is also important to consider cultural differences between sites, for example the name of the patient's parent or caregiver is a required field in the registry, but in some countries, it is considered inappropriate to ask for parents' first names.

### 3.3 Facilitators

Facilitators to the implementation of the SJCARES registry were identified through interviews with participants and are described below. Quotes from participants are included in Table 2.

#### 3.3.1 High internal support and motivation

Administrators were motivated by various factors to implement the SJCARES registry at their respective sites, including opportunities for future research. Clinicians explained how the registry allows them to assess treatment options and conduct quality improvement projects to improve care for their patients.

#### 3.3.2 Dedicated support staff

Clinicians are burdened by other work responsibilities, and a dedicated staff member is necessary to complete registry tasks. If there are adequate staff, clinicians noted that they can rely on them to compile information and communicate directly with the administration when needed. Having a team of support staff specialized in pediatric oncology makes implementation easier as they are already familiar with the specific information required by the registry.

#### 3.3.3 Clear and extensive training courses

Various participants reported that completing the training is a barrier to implementation due to lack of time and personnel. However, once completed, participants said that having the clear and extensive training courses helped implement the SJCARES registry. The online training course provides foundational information for both data entry specialists and clinicians on effectively utilizing the registry.

#### 3.3.4 External support

Support from external departments and organizations was noted to facilitate the adoption of the registry. For example, one center was encouraged to use the registry by their national society of pediatric oncology, reinforcing the societal advantage of participating in cancer registries, and subsequently inspiring local administrators' support. Participants noted that support from administrators at their site, particularly by hiring staff and providing additional resources and guidance, helps ensure data entry into the registry is ongoing and meets the desired objectives.

#### 3.3.5 Registry is easy to use

The registry is well‐organized, has clear data‐sharing procedures, and is easy to navigate. Because of the practical and accessible nature of the registry, user onboarding is relatively straightforward.

#### 3.3.6 Good organization of medical records

A few participants described how having well‐organized records rooms makes it easier to extract relevant patient information into the registry. Documentation at the time of admission provides important information that is necessary to enter into the registry.

### 3.4 Recommendations

Participants also offered several recommendations that mitigate barriers to implementation. Quotes from participants are included in Table 4.

**TABLE 4:** Recommendations for registry implementation from participants.

Recommendation	Frequency (*N* = 18)	Illustrative quotes
Completion of training	4	“…good to be familiarized with the patients, and the information that needs to be inputted…they just need to gather more information about their patients so that…when they will be implementing the use of the registry, it will be a lot more easier for them.” [Data Clerk 02] “My suggestions for other hospitals want to use this database is that before they actually—before they use this database, the training is very important because there still are some expression … or cultural difference between these countries” [Administrator 07, Data Clerk 04]
Dedicated personnel and support of staff	3	“I think they need two staff, the dedicated data entry specialist and an MD monitor. Also, the support of all the staff who help in collecting the data and giving to the data specialty. Because sometimes the data entry specialist is not the one who is taking round of whole hospital with the cancer patient and fill the form himself…To run this smoothly, I think they have to convince all the member of team that how significant it is, and it's a shared property. It's not one person's property.” [Clinician 06]
Communication among staff	3	“I think they should realize that this is like shared data and everybody has the right to, because all the consultants who are working in [center] have their own way of collecting data. Nobody's telling them, do not collect this data, do not take this data. They are patients. They are registered. There are computers. They can have their own data, their collection, their research. Nobody's putting any hurdle on them.” [Clinician 06]
Proper incentives	2	“Resources is very important because nobody will do such a hard work without incentives and without proper salary. I think in low‐middle income country, this is one of the biggest, biggest challenge, to run the registries.” [Clinician 06]
Familiarity with information in registry	1	“…good to be familiarized with the patients, and the information that needs to be inputted…they just need to gather more information about their patients so that…when they will be implementing the use of the registry, it will be a lot more easier for them.” [Data Clerk 02]
Offline back‐up	1	“Then the next one would be, well, to always have a back‐up, like an offline back‐up.” [Data Clerk 05]
Proof of data	1	“If you don't have data, you can't prove to the world that you are doing anything. Nobody will trust you can do the great work, treat patients. If you don't prove it with a number, you don't do anything. We need to prove to the world that this is what we are doing, and not only to prove to the world that we are doing something. We need to be—we need to bring out information in a standardized way so that conclusion can be drawn and recommendation.” [Clinician 11]

*Note*: Participants also offered several recommendations that both mitigate barriers and leverage facilitators mentioned in Table 2. *N* denotes the frequency each recommendation was mentioned in interviews with participants.

#### 3.4.1 Proper incentives

Participants emphasized that proper incentives are needed to ensure the registry will be maintained. Personnel working with the registry are hesitant to take on more responsibilities without being appropriately compensated. Participants described the importance of having access to resources to provide proper financial incentives and salaries to those participating in the registry.

#### 3.4.2 Dedicated personnel and support of staff

A few participants proposed having a dedicated data clerk to input data into the registry. This support is needed to capture all patients in the pediatric oncology department. Having adequate support from staff in the hospital would prevent burnout from the data entry specialists and provide more dedicated time to spend entering data.

#### 3.4.3 Awareness of cultural differences

Participants advised that certain language used in the registry may be misunderstood due to differences in culture and nomenclature. As a result, a portion of the training should focus on ensuring data clerks understand the registry's terminology.

#### 3.4.4 More clear instructions and oversight from St. Jude

A few participants thought that St. Jude should provide more clear instructions on how to enter data and be more directly involved in the training before sites start actively collecting data.

#### 3.4.5 Completion of training

Participants across all groups suggested that completing all the training modules will significantly facilitate registry implementation. Data clerks also emphasized having training tailored to caring for patients with low health literacy levels helps facilitate data collection for these patients.

#### 3.4.6 Communication among staff

Several participants advocated for more communication between data clerks and clinicians to ensure better understanding and standardization of the data.

## 4 DISCUSSION

Although HBCRs are identified within the GICC *CureAll* framework as an integral tool to support comprehensive childhood cancer control, no data on the unique barriers and facilitators to successful implementation exist. This is the first study to systematically explore these factors.

Barriers we discovered in our study such as accessing additional resources and obtaining support from leadership are consistent with barriers reported in previous literature in relation to the difficulty of obtaining and sustaining resources, and obtaining external departmental support for advanced healthcare services in LMICs.^11^, ^16^, ^17^ However, due to the multicenter nature of the SJCARES registry, cultural considerations and language barriers were unique barriers found in our study. This finding demonstrates the need to develop individualized work processes and training to facilitate cancer registry development and sustainability.^17^ Barriers to implementing the SJCARES registry were consistent across all sites regardless of phase of implementation. There was no strong correlation between specific barriers encountered by participants and phase of implementation. This lack of correlation suggests the ubiquity of these challenges to all phases of registry implementation. Sites which had progressed to further stages of implementation had more insight into facilitators to using the registry, while participants at sites still in the adoption phase had less to report on factors which made their work easier. Current known literature has been able to assess the implementation of cancer registries in individual countries, providing simplified processes to create individualized recommendations to optimize workflow, increase data quality, and increase the capacity to build a sustainable registry.^11^, ^17^ As a result, regular workflow analyses are necessary for centers to develop optimized work processes to overcome the cultural considerations and language barriers encountered to help facilitate the implementation of the registry.

Our data are critical to begin developing interventions that can support the successful implementation of HBCRs as recommended by the GICC 4th pillar of evaluation and monitoring. In particular, our decision to utilize the CFIR framework to evaluate barriers and facilitators will enhance the generalizability of our findings. CFIR is now a commonly used tool in providing a systematic approach to eliciting the key determinants of implementation. It is highly adaptable to different contexts, and the version utilized in this study has been contextualized to fit the needs of LMICs.^7^ Built on over of decade of prior implementation science frameworks, our goal in using this tool was to ensure our results are robust. By formally conducting a CFIR analysis and linking the results to the ERIC project, our study identified evidence‐based missed opportunities during the pre‐study implementation process. These data provide a blueprint for the SJCARES team to refine existing implementation processes in a more holistic manner by layering in complementary interventions to tackle the identified barriers (Table 3).

The interview guide and analytic approach described in this study will provide other researchers with a transparent means to replicate our work in research and practice. Although we included a diverse group of registry teams, with representation from a spectrum of regions and income levels, additional contextual barriers will exist. Thus, it is critical that others understand the barriers and enablers in their local context. Using the materials generated through this study can support rapid local evaluation and design of local mitigation strategies.

Non‐responsiveness, language barriers and difficulty coordinating translation services posed a challenge to the interviews. Data collection continued until we reached data saturation, defined as the point when no new information is elicited through subsequent interviews. We estimated this number to be between 20 and 25 participants. However, data saturation was not met due to challenges with establishing contact with a few potential interviewees, which resulted in a limited quantity of interviews being conducted. Despite not reaching data saturation, the results still display significance in the implementation of the SJCARES registry. Sites that were unable to conduct interviews in English were excluded, as well as sites experiencing political unrest. There were also challenges establishing contact with potential participants that resulted in limited interviews. The subjective nature of the data and ambiguity of language may leave responses open to misinterpretation. For participants whose native language was not English, some difficulty understanding questions and responses occurred, however the semi‐structured format of the interviews allowed us to rephrase questions that participants did not understand, and ask follow up questions to assist the participant in providing the requested information. Also, many of the barriers that were discovered to prevent successful SJCARES implementation (i.e., lack of resources, poor internet connection, language barriers, cultural differences, other work responsibilities) may have also hindered additional sites from participating in this study. Despite these limitations, our data provide valuable insight into the challenges of registry implementation in resource‐limited settings. Further study areas include formally testing proposed strategies to address implementation barriers and exploring additional barriers and facilitators in more contexts.

## 5 CONCLUSION

Our findings are the first step in remedying issues faced during onboarding and implementing HBCRs in LMICs. Key barriers and facilitators discovered through these interviews can further guide understanding of why the speed and success of implementation of the SJCARES registry has been slow, and how this process can be improved. This will allow us to effectively develop a HBCR implementation approach that is sustainable, fiscally feasible, and adaptable. Optimization of HBCR implementation is aligned with recommendations from the WHO GICC and can significantly enhance the quality of evaluation and monitoring of pediatric oncology services in many countries.

## AUTHOR CONTRIBUTIONS

**Melissa R. Maas:** Data curation (equal); formal analysis (equal); investigation (equal); writing – original draft (equal); writing – review and editing (equal). **Allison Yang:** Data curation (equal); formal analysis (equal); investigation (equal); writing – original draft (equal); writing – review and editing (equal). **Michele A. Muir:** Conceptualization (equal); funding acquisition (equal); methodology (equal); supervision (equal). **James B. Collins IV:** Funding acquisition (equal); methodology (equal); supervision (equal). **Courtney Canter:** Formal analysis (equal); project administration (equal). **Gevorg Tamamyan:** Investigation (supporting). **Inam Chitsike:** Investigation (supporting). **Francine Kouya:** Investigation (supporting). **Kim Hoa Nguyen:** Investigation (supporting). **Alia Ahmad:** Investigation (supporting). **Ana Patricia Alcasabas:** Investigation (supporting). **Yi‐Jin Gao:** Investigation (supporting). **Kim Johnson:** Funding acquisition (equal). **Gia Ferrara:** Methodology (supporting). **Nickhill Bhakta:** Conceptualization (equal); funding acquisition (equal); methodology (equal); project administration (equal); resources (equal); software (equal); supervision (equal); validation (equal); visualization (equal); writing – review and editing (equal). **Benyam Muluneh:** Conceptualization (equal); funding acquisition (equal); methodology (equal); project administration (equal); supervision (equal); validation (equal); visualization (equal); writing – review and editing (equal).

## FUNDING INFORMATION

This study was approved by the both the University of North Carolina, and St Jude Children's Research Hospital Institutional Review Boards (#22‐0226). All study participants were required to provide signed written informed consent to partake in the study. This work was supported by the St. Jude‐Wash U Implementation Science Collaborative and UNC Chapel Hill CTSA grant UL1TR002489.

## Supporting information


**Data S1:** Supporting Information.

## Data Availability

Data is available upon request from the authors.
