# Supplementary material for: Evaluating implementation of a hospital‐based cancer registry to improve childhood cancer care in low‐ and middle‐income countries
Source: Cancer Med. 2024 Sep 9;13(17):e70125. doi: 10.1002/cam4.70125 (PMC11382012; doi:10.1002/cam4.70125)
Supplement: Supplementary file 1 — Data S1: Supporting Information. [file CAM4-13-e70125-s001.docx]

**SJCARES Registry Semi-Structure Interview Guide (*Implementation*)**

**Intro**: **“Hi my name’s ______ and thank you for meeting with me today. As a brief reminder, the purpose of this interview is to gather your thoughts and opinions surrounding the implementation of the SJCARES Registry at your hospital. Although you may already be familiar, the SJCARES Registry is a cloud-based hospital cancer registry developed by St. Jude that documents quality patient data for children with cancer.** Our conversation today will be kept confidential, with all identifying information about you, like your name, removed. Participation in this interview is voluntary - please feel free to skip any question that you do not wish to answer, and you can end the interview at any time. **Before we begin, I just want to confirm – Do you agree to participate in the interview? And are you okay being recorded? – Thank You”**

To start off, we’d like to know a little more about you with demographic information:

- What is your profession? What role do you currently have in the center/hospital/institution?
- How many years have you been working in this center/hospital/institution?
- What is your role with the SJCARES Registry?

Thanks, now we would like to start with the interview questions; we are going to talk about quality improvement in your center and questions related to the implementation of the SJCARES Registry:

1. Before the SJCARES Registry, what other forms of a registry existed in your hospital?
   1. Have you ever participated in registry documentation before the SJCARES Registry?
2. Before the SJCARES Registry, what collaborative projects, if any, has your hospital participated in with other centers (hospitals/organizations)?
   1. Have you participated in collaborative projects with other centers before the SJCARES Registry?
3. In general, to what extent are new ideas used to improve patient care in your hospital?
   1. How do administrators and influential leaders respond to initiatives proposed for quality improvement in your hospital?

We are going to talk about the *adoption and* *implementation* of the SJCARES Registry in your center. As a reminder, adoption is the onboarding of the registry and implementation is the data collection phase. Don’t worry if you cannot answer some questions.

1. Why did your hospital decide to *adopt and* *implement* the SJCARES Registry?
   1. How strong, or not strong, was the need to *implement* the SJCARES Registry? Could you explain? Why?
2. Please describe what steps have been accomplished to *implement* the SJCARES Registry in your hospital.
3. Were there sufficient resources (e.g., financial, technology, personnel) for the *implementation* of the SJCARES Registry (Phase: onboarding, pilot, data collection, data utilization)?
   1. If [yes], what were they and how did you obtain them?
   2. If {no}, did you obtain the necessary resources?
      1. If [yes], what were they and how did you obtain them?
   3. How do you foresee the continuity of resources in the future?
4. What training, if any, did you receive on the SJCARES Registry?
   1. How satisfied were you with this training? What, if anything, was missing from the training? What else would you have liked to have had as part of the training?
5. How confident are you that you will be able to successfully adopt and implement the SJCARES Registry?
   1. What gives you that level of confidence or lack of confidence?
6. How did your hospital plan the *adoption and implementation* of the SJCARES Registry?
   1. How did you all communicate about the SJCARES Registry to members of the personnel or departments of the hospital?
7. What type of changes or adaptations, if any, were necessary to the SJCARES Registry to facilitate the *implementation* of the registry in your hospital? (e.g., omissions, requests for changes)
8. What type of changes or adaptations, if any, were necessary to your work processes to facilitate *implementation* of the SJCARES Registry in your hospital?
9. Beyond the leaders of the SJCARES Registry, who else had an important role in the *adoption and implementation*?
   1. How did you manage to involve people in your hospital to participate in the SJCARES Registry?
10. What factors facilitated or made easier the *implementation* of the SJCARES Registry in your hospital?
11. What barriers did you or your hospital encounter during the *implementation process* of the SJCARES Registry?
    1. How, if at all, did you address these barriers?
12. What level of participation have the directors / leaders of your hospital had in the *implementation* of the SJCARES Registry?
    1. How does hospital leadership perceive the SJCARES Registry?
13. Was there anyone outside of the hospital who helped with the *adoption and* *implementation* of the SJCARES Registry? If yes, could you describe?
    1. What external factors, if any, affected the decision to implement the SJCARES Registry in your hospital? (local, state, national)
    2. What other considerations, if any, (financial, other incentives) contributed to the decision to *implement* the SJCARES Registry in your hospital?
14. What recommendations or advice would you give to a center that wants to *implement* the SJCARES Registry?
    1. How well do you think other hospitals will be able to *adopt and implement* the SJCARES registry?
15. How do you foresee the utilization of the registry at your hospital over the next 5 years?
16. Do you have any other comments about the *implementation* of the SJCARES Registry in your hospital that we didn’t cover in this interview or anything else you learned?

**“Thank you for your thoughtful feedback. We will share our findings to participants at the conclusion of this study. Our goal is to use this information to design a sustainable SJCARES Registry with clear implementation strategies.”**

**SJCARES Registry Semi-Structure Interview Guide (*Adoption*)**

**Intro**: **“Hi my name’s ______ and thank you for meeting with me today. As a brief reminder, the purpose of this interview is to gather your thoughts and opinions surrounding the implementation of the SJCARES Registry at your hospital. Although you may already be familiar, the SJCARES Registry is a cloud-based hospital cancer registry developed by St. Jude that documents quality patient data for children with cancer.** Our conversation today will be kept confidential, with all identifying information about you, like your name, removed. Participation in this interview is voluntary - please feel free to skip any question that you do not wish to answer, and you can end the interview at any time. **Before we begin, I just want to confirm – Do you agree to participate in the interview? And are you okay being recorded? – Thank You”**

To start off, we’d like to know a little more about you with demographic information:

- What is your profession? What role do you currently have in the center/hospital/institution?
- How many years have you been working in this center/hospital/institution?
- What is your role/what will your role be in the SJCARES Registry?

Thanks, now we would like to start with the interview questions; we are going to talk about quality improvement in your center and questions related to the implementation of the SJCARES Registry:

1. Before the SJCARES Registry, what other forms of a registry exist in your hospital?
   1. Have you ever participated in registry documentation before the SJCARES Registry?
2. Before the SJCARES Registry, what collaborative projects has your hospital participated in with other centers (hospitals/organizations)?
   1. Have you participated in collaborative projects with other centers before the SJCARES Registry?
3. In general, to what extent are new ideas used to improve patient care in your hospital?
   1. How do you believe that administrators and influential leaders, respond to initiatives proposed for quality improvement in your hospital?

We are going to talk about the *adoption* of the SJCARES Registry in your center. As a reminder, adoption is the onboarding of the registry and implementation is the data collection phase. Don’t worry if you cannot answer some questions.

1. Why did your hospital decide to *adopt* the SJCARES Registry?
   1. Was there a strong need to *adopt* the SJCARES Registry? Why?
2. Please describe what steps have been accomplished to *adopt and onboard* personnel on the SJCARES Registry in your hospital.
3. Were there sufficient resources (e.g., financial, technology, personnel) for the *onboarding phase* of the SJCARES Registry?
   1. If [yes], what were they and how did you obtain them?
   2. If {no}, did you obtain the necessary resources?
      1. If [yes], what were they and how did you obtain them?
   3. How do you foresee the continuity of resources in the future?
4. What training have you received on the SJCARES Registry?
   1. Was this training enough? Was there anything missing? What else would you have liked to have had as part of the training?
5. How confident are you that you will be able to successfully adopt and implement the SJCARES Registry?
   1. What gives you that level of confidence or lack of confidence?
6. How did your hospital plan for the *adoption process* of the SJCARES Registry?
   1. How did you all communicate about the SJCARES Registry to members of the personnel or departments of the hospital?
7. What type of changes or adaptations were necessary to the SJCARES Registry to facilitate the *adoption* of the registry in your hospital?
8. What type of changes or adaptations were necessary to your work processes to facilitate the *adoption* of the SJCARES Registry in your hospital?
9. Beyond the leaders of the SJCARES Registry, who else had an important role in the *adoption and onboarding*?
   1. How did you manage to involve people in your hospital to participate in the SJCARES Registry?
10. What factors facilitated or made easier the *onboarding process* of the SJCARES Registry in your hospital?
11. What barriers did you or your hospital encounter during the *onboarding process* of the SJCARES Registry?
    1. How did you overcome these barriers?
12. What level of participation have the directors / leaders of your hospital had in the *adoption and onboarding* of the SJCARES Registry?
    1. How does hospital leadership perceive the SJCARES Registry?
13. Was there anyone outside of the hospital who helped with the *adoption or onboarding* of the SJCARES Registry?
    1. Was there any external factor (local, state, national) or other considerations (financial, other incentives) that contributed to the decision to *adopt* the SJCARES Registry in your hospital?
14. What recommendation or advice would you give to a center that wants to *adopt* the SJCARES Registry?
    1. How well do you think other hospitals will be able to adopt and implement the SJCARES registry?
15. How do you foresee the utilization of the registry at your hospital over the next 5 years?
16. Do you have any comments about the *adoption or onboarding process* of the SJCARES Registry in your hospital that we didn’t mention in this interview or anything else you learned?

**“Thank you for your thoughtful feedback. We will share our findings to participants at the conclusion of this study. Our goal is to use this information to design a sustainable SJCARES Registry with clear implementation strategies.”**
